# Burden of death and disability due to adverse effects of medical treatment in India: An analysis using the global burden of disease 2019 study data

**DOI:** 10.1016/j.heliyon.2024.e24924

**Published:** 2024-01-18

**Authors:** Ashwin Kamath, Sahana D. Acharya, Poovizhi Bharathi R

**Affiliations:** Department of Pharmacology, Kasturba Medical College, Mangalore, Manipal Academy of Higher Education, Manipal, India

**Keywords:** Adverse effects, Pharmacotherapy, Death, Disability, Disease burden

## Abstract

Unsafe patient care can result in an adverse event that may lead to hospitalization, disability, or death. India has a vast and diverse population with varying degrees of access to tertiary healthcare. However, there is a lack of studies analyzing the burden of healthcare-related adverse events. We aimed to determine the burden of adverse effects of medical treatment (AEMT) in India from 2010 to 2019 using the global burden of disease (GBD) 2019 study database. Using the GBD data, we computed estimates for deaths and disability-adjusted life years (DALY) due to AEMT at the national level and stratified them based on age and gender. AEMT contributed to less than 0.01 % of death and DALY rates due to all causes in India. From 2010 to 2019, there was a decrease in the death rate from 2.34 (1.75–2.66) to 2.33 (1.73–2.86) per 100000 population. The number of deaths and DALYs was highest in the 50–74-year age group and in females. There has been a decrease in the death and DALY rates in India over the past decade. AEMT accounts for only a small percentage of deaths due to all causes; however, the potential underreporting and the impact of medical treatment-related adverse events on the public perception regarding healthcare services need to be studied.

## 1 Introduction

The ethical principle of healthcare ‘to do no harm’ includes the undertaking of measures by health professionals to prevent any treatment-related avoidable harm to patients. Unsafe patient care can result in an adverse event that may lead to hospitalization, disability, or death [1]. Even in the absence of a serious outcome, adverse events can undermine a patient's faith in the treatment or healthcare provider, especially in the context of a lack of adequate doctor-patient communication. In low-to-middle-income countries, it is estimated that safety lapses result in 134 million adverse events, causing 2.4 million deaths, each year [2]. A systematic review of the incidence and nature of adverse events in hospitalized patients showed that the median incidence was 9.2% (interquartile range [IQR], 4.6%–12.4%), and 7.4% of the adverse events caused the death of the patients [3].

A meta-analysis conducted on preventable patient harm across medical settings including hospitals, various specialties, and primary care showed a 6% prevalence for preventable harm, and 12% (IQR, 9%–15%) of these caused disability or death [4]. Drug management incidents and other therapeutic management incidents accounted for the highest proportion of preventable patient harm, followed by incidents related to surgical procedures, healthcare infections, and diagnosis [4]. Data from the Department of Health and Human Services (DHHS), United States of America, shows that treatment-related adverse events persist to be a significant problem despite the adoption of mitigating measures [5]. Moreover, medical treatment-related adverse events are yet to be considered a significant public health problem in many countries where efforts are currently directed toward tackling the growing burden of non-communicable diseases. A basic step toward addressing the issue of adverse effects of medical treatment (AEMT) is to determine the magnitude of the problem within a geographic context. In view of improving the standard of care in the health sector, the Government of India established the National Accreditation Board for Hospitals & Healthcare Providers, a constituent of the Quality Council of India [6]. Certain surveillance systems, such as the Adverse Events Following Immunization and Pharmacovigilance Program of India, which monitor adverse events following immunization and drug treatment, respectively, help to scrutinize the cause, nature, and incidence of adverse events at the national level [7]. However, there is a lack of studies describing the burden of adverse effects specifically occurring due to or in relation to medical treatment in the Indian population. Vital statistics published annually at the state and national levels provide useful data in this regard. More recently, the National Patient Safety Implementation Framework (2018–2025) was implemented by the Ministry of Health and Family Welfare, Government of India, with one of the objectives being to assess the nature and scale of adverse events in healthcare and establish a system of reporting and learning [8]. In a larger geographic context, the Global Burden of Diseases (GBD) study is an international, multi-collaborator effort that collects data from various sources, such as government-published vital statistics as well as data from published literature, to generate country-specific data for various causes of disability and mortality [9]. Our study aimed to determine the burden of AEMT in India for the period ranging from 2010 to 2019 using the GBD 2019 study database.

## 2 Methods

We conducted a retrospective, descriptive study of AEMT for the period 2010 to 2019 reported from India and available in the GBD database. The GBD 2019 study provided estimates (incidence, prevalence, deaths, disability-adjusted life years [DALY]) for the period 1990–2019 for various causes of death based on age, gender, country, regions and super-regions, and subnational level for some countries [9]. The causes for various diseases and injuries were organized in a hierarchical framework moving from the broadest level-1 causes (communicable diseases, non-communicable diseases, and injuries) to the specific level-4 causes (301 causes). The cause of interest in the current study was the level 3 cause ‘Adverse effects of medical treatment.’ [9] This cause constitutes death or disability due to exposure to medical treatment or procedures, irrespective of whether this occurs within or outside a hospital setting. DALYs equal the sum of years of life lost and years lived with disability. One DALY equals one lost year of healthy life [10]. The data were obtained from various sources such as census, disease registries, vital statistics available from governmental websites, published reports, health survey reports, etc. Since the data may have been incomplete or not available for all states and all years, rather than excluding these regions and time points, the GBD process generated estimates based on data modeling, which considered any available data for that region in any of the time points and the possible influence of covariates to generate an approximate estimate [11]. The basis for generating such modeled estimates, rather than reporting no estimates, was that the GBD principles consider that modeled estimates provide at least a rough guide for policymakers in assessing the burden of the problem, which would otherwise be likely ignored in the absence of any supporting data on estimates.

The data on AEMT obtained from various sources were coded using the International Classification Diseases code, ICD-9 and ICD-10 [9]. The GBD study process relied on a collection of these codes to identify cases of death due to AEMT. Specifically, the cause ‘Adverse effects of medical treatment’ included the following ICD-10 codes in the GBD study process: N30.4, Y40–Y84.9, and Y88–Y88.3 (Supplementary Table 1).

Using the GBD data, we obtained estimates for incidence, deaths, and disability due to AEMT (https://vizhub.healthdata.org/gbd-results/) [12]. The estimates were determined at the national and state levels and stratified based on age and gender. To determine the difference between the reported estimates and those generated by the GBD modeling process, we compared the yearly mortality rate at the national level and for the state of Karnataka as derived from the GBD database and the medical certification of cause of death (MCCD) report published by the Office of the Registrar General & Census Commissioner, India [13]. The latter is based on reports from medical professionals from select hospitals, mostly confined to urban areas, of the various states/union territories, and hence, comprises only a fraction of the total registered deaths. The ICD-10 codes for AEMT in the MCCD reports differ from the GBD codes in that they exclude N30.4 and Y88–Y88.3. We also compared the GBD estimates for India with those of the United States of America for 2019 because the latter has estimates based on robust data coverage in the GBD database [9]. A comparison was also made based on the sociodemographic index (SDI) categories as described by the GBD study process [14]. SDI is a composite indicator based on the total fertility rate under the age of 25 years, mean education among those aged ≥15 years, and the lag distributed income per capita (GDP per capita which is a 10-year lagged average to smooth out the short-term jumps and drops); it correlates with the health outcomes for a country [14]. There are five SDI country categories: high, high-middle, middle, low-middle, and low SDI. India is included in the low-middle SDI category.

The study protocol was approved by the Kasturba Medical College Institutional Ethics Committee (IEC KMCMLR-12/2022/497).

### 2.1 Statistical analysis

Estimates (incidence, deaths, DALY numbers and rates) for AEMT in India (total and stratified by age and gender), the United States, and based on SDI categories were downloaded from the GBD database (https://vizhub.healthdata.org/gbd-results/) as Microsoft.csv files (Microsoft Corporation, Redmond, Washington, United States). The data were then exported to a Microsoft Excel worksheet (Microsoft Corporation, Redmond, Washington, United States) for further analysis and visualizations were generated. Rates are expressed per 100000 person-years, and the estimates are age-standardized. The change in the estimates from 2010 to 2019 has been reported. The point estimates along with 95% uncertainty intervals are presented. An uncertainty interval consists of a range of values that is likely to contain the true estimate for the population; this is obtained by sampling 1000 draws, and the 2.5th and 97.5th quantiles of all draws form the uncertainty interval [9]. Data from the MCCD reports were manually entered into a Microsoft Excel file.

## 3 Results

### 3.1 AEMT incidence, mortality, and disability at the national level

**Fig. 1:**
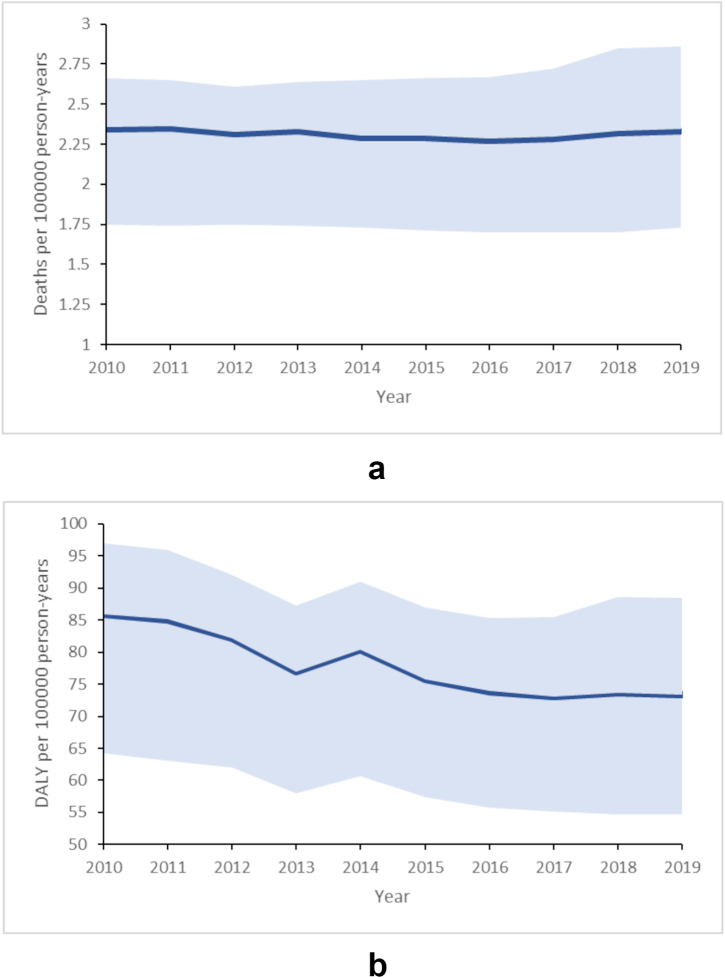
Change in **a)** death and **b)** DALY rates due to adverse effects of medical treatment from 2010 to 2019. Rates are age-standardized and expressed per 100000 person-years. The dark blue solid line represents the point estimate, and the light blue shaded areas on either side of the line represent the 95% uncertainty interval. DALY, disability-adjusted life years.

The incidence rate of AEMT in India was 140.89 (114.03–167.92) and 162.40 (131.14–195.95) per 100000 person-years in 2010 and 2019, respectively. In 2019, 32464 (24076–39747) deaths were reported due to AEMT; the corresponding number in 2010 was 28830 (21491–32707). In terms of the death rate, this constituted a decrease from 2.34 (1.75–2.66) to 2.33 (1.73–2.86). Regarding DALYs, a decrease was seen both in absolute numbers (2010–2019, 1058375 [792000–1194384] to 1020453 [761309–1230656]) and the DALY rate (2010–2019, 85.95 [64.32–97.00] to 73.38 [54.74–88.49]). The country-level year-wise change in the death and DALY rates is shown in Fig. 1a and b, respectively. In terms of death and DALY rates due to all causes, those due to AEMT constituted less than 0.01%. The GBD data source includes MCCD reports from 2009 to 2012, Odisha MCCD reports from 2009 to 2013, Karnataka MCCD reports from 2014 to 2015, and Delhi MCCD report for 2013.

### 3.2 AEMT burden based on age

In 2019, the major burden of deaths was among those aged more than 50 years (Supplementary Table 2), with the highest absolute number of deaths being in the 50–74 years age group (14877 [10684–18666]), while the highest death rate was among those aged ≥75 years (29.71 [21.86–37.10]). Absolute DALY was highest in the 50–74 years age group (388668 [278699–490352]), whereas the highest DALY rate was in those aged ≥75 years (243.10 [178.48–304.38]). Death and DALY numbers and rates in each age group are presented in Supplementary Table 2.

### 3.3 AEMT burden based on gender

**Fig. 2:**
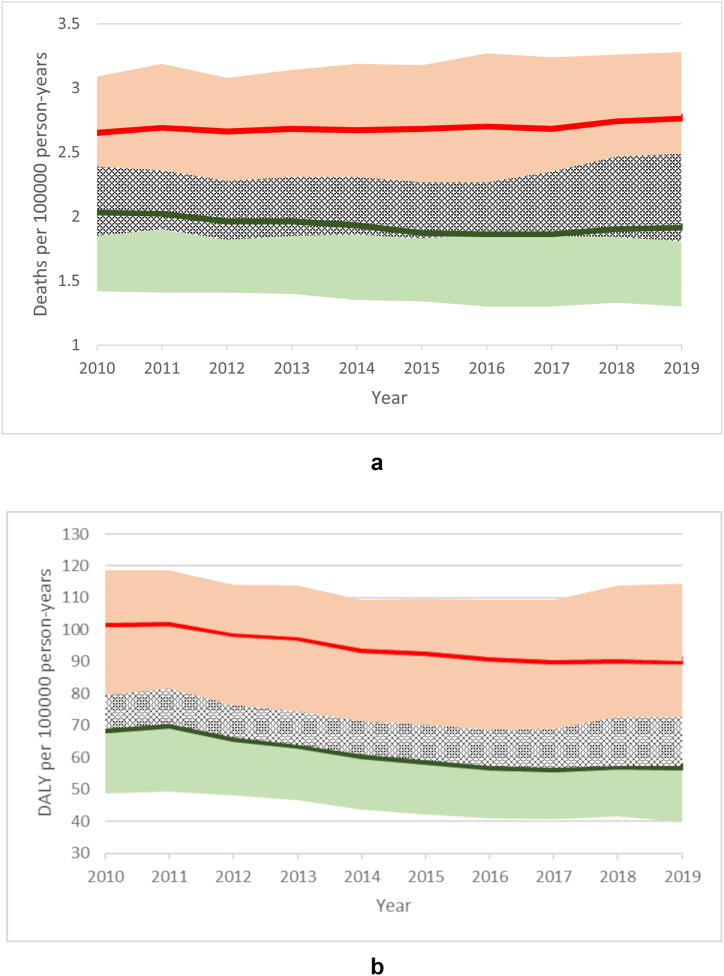
**a)** Death and **b)** DALY rates due to adverse effects of medical treatment in males and females. Rates are age-standardized and expressed per 100000 person-years. The green and red solid lines represent the point estimates in males and females, respectively. The light green and light red shaded areas represent the 95% uncertainty intervals for males and females, respectively, and the dotted area represents an overlap between the uncertainty intervals for males and females. DALY, disability-adjusted life years.

The burden of AEMT was higher among females in terms of death and DALY numbers (Supplementary Table 3) and rates (Fig. 2a and b). Females constituted 58% of the total number of deaths in 2019. The number of deaths in males increased from 12967 (8997–15161) in 2010 to 13704 (9260–17771) in 2019; in females, the number increased from 15862 (11051–18625) to 18760 (12244–24263). DALYs in males decreased from 444344 (313297–512253) to 408139 (282433–517872), and in females, from 620050 (430890–719537) to 612314 (406869–774798). Fig. 2a and b shows the year-wise changes in death and DALY rates, respectively, for both genders. Although the death rates did not decrease, a downward trend was observed in DALY rates in both genders.

The incidence rate was higher in males than in females, and an increase in the rate from 2010 to 2019 was observed in both genders. In males, the incidence rate increased from 151.50 (120.13–184.73) in 2010 to 167.52 (132.71–205.68) in 2019; in females, the increase was from 129.64 (105.58–156.26) to 157.01 (126.85–188.72) per 100000 person-years.

### 3.4 Mortality and disability due to AEMT based on SDI categories

**Fig. 3:**
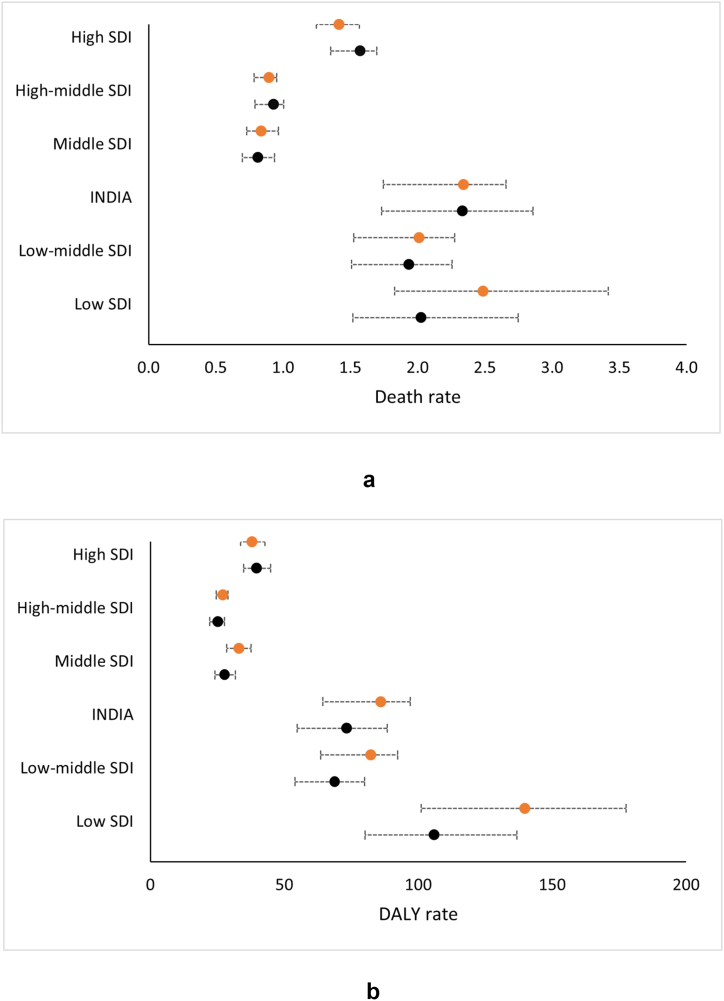
Comparison of **a)** death and **b)** DALY rates due to adverse effects of medical treatment in India with those of other countries based on sociodemographic index (SDI) categories. Orange circles represent data for 2010; black circles represent data for 2019. The dotted horizontal lines represent the 95% uncertainty intervals. Rates are age-standardized and expressed per 100000 person-years. DALY, disability-adjusted life years.

The death and disability rates in India have been compared with the estimates for various SDI categories in Fig. 3a and b, respectively. The death rate was higher than the average estimate for low-middle SDI countries and higher than that for the middle SDI countries and above. The death rate was lower than that in the low SDI countries in 2010 but not 2019. A similar trend was observed with regard to the DALY rates, with the exception that the rates in 2010 and 2019 were lower than those of the low SDI countries.

Table 1 shows the estimated mortality numbers based on the GBD modeling framework and the number of deaths due to AEMT reported in the annual report on the medical certification of cause of death published by the Office of Registrar General, India. A large difference in the reported and estimated numbers is seen, the difference ranging from 10 to 200 times. The same was true on inspecting the data for the state of Karnataka.

**Table 1:** Number of deaths due to adverse effects of medical treatment based on GBD 2019 study and MCCD report.

Year	Number of deaths due to AEMT			
	In India		In Karnataka	
	GBD	MCCD	GBD	MCCD
2010	28830	142	1108	8
2011	29416	584	1159	14
2012	29382	489	1185	39
2013	30018	1209	1234	NA
2014	29963	556	1255	16
2015	30284	700	1291	8
2016	30977	870	1329	16
2017	30470	851	1348	7
2018	31935	1469	1374	19
2019	32464	3245	1365	10

AEMT, adverse effects of medical treatment; GBD, global burden of disease; MCCD, medical certification of cause of death.

The GBD estimates of AEMT for India and the United States of America for 2019 are compared in Table 2. Although the death and DALY rates were higher in India, the incidence and prevalence rates were higher in the United States in 2019. In addition, a decrease in the death rate was observed in India compared with that in 2010, whereas an increase was observed in the United States. Supplementary Table 4 shows the GBD estimates for other countries.

**Table 2:** Comparison of AEMT statistics in India and the United States for 2019.

Variable^a^	India	United States of America
Incidence rate	162.40 (131.14–195.95)	1934.95 (1594.02–2356.44)
Prevalence rate	12.41 (9.05–16.18)	147.35 (108.78–190.68)
Death (Number)	32464 (24076–39747)	5016 (4556–6232)
Death rate	2.33 (1.73–2.86)	1.53 (1.39–1.9)
Percentage of all deaths	0.35 (0.26–0.41)	0.17 (0.15–0.21)
DALY rate	73.38 (54.74–88.49)	53.83 (45.34–65.79)
% change in death rate (2010–2019)	−0.033%	1.21%

AEMT, adverse effects of medical treatment; DALY, disability-adjusted life years.

a Rates are expressed per 100000 person-years.

## 4 Discussion

Our study showed an increase in the incidence rate of AEMT in India from 2010 to 2019. While there was also an increase in the absolute number of deaths due to AEMT, the death and DALY rates decreased over this period. AEMT accounted for less than 0.01 % of the disease burden due to all causes in India. In terms of age, the highest death rate was among those aged ≥75 years although the absolute number was highest in the 50–74-year age group. Females had a higher disease burden than males, with death rates increasing in the former. Although the incidence rate was higher in males, the year-on-year increase was higher in females. Earlier studies based on the GBD data conducted in other countries show inconsistent results with regard to gender; while the study by Lunevicius et al. in the United Kingdom found a higher burden of the problem in males [15], no significant gender difference was noted by Sunshine et al. in the United States [16]. The death and DALY rates due to AEMT in India were higher than the average for low-middle SDI countries in 2019.

To the best of our knowledge, this is the first study to describe the burden of AEMT in India and to compare the data with the numbers reported in the MCCD reports. Nauman et al. described the AEMT burden globally using GBD 2017 data [17]; however, we felt it important to not only present the latest India-specific data but also describe the underlying data source and compare it with other countries to understand the magnitude of the problem. On comparison based on SDI, both death and DALY rates were higher in India than in the middle, high-middle, and high SDI countries. The rates were also higher than the average for the low-middle SDI category to which India belongs. Compared with 2010, the death rate increased in high-middle and high SDI countries, with the latter also showing an increase in the DALY rate; these rates showed the highest decrease in the low SDI countries.

A few studies in India have quantified adverse drug reactions (ADR) as a cause of hospitalization/death. A study conducted in an internal medicine ward of a hospital in Gujarat reported ADR as a cause for hospitalization in 5.42% (47/830) of the study sample; of these, 4.25% were fatal, 29.79% were life-threatening, and 62.83% necessitated hospitalization or its prolongation [18]. In a study of 6026 pediatric patients admitted over 1 year in Maharashtra, ADRs occurred in 182 (incidence, 3.02%); 25 ADRs (13.7%) were severe, and 2 had fatal outcomes [19]. In a study of 1033 patients at a North Indian tertiary care hospital, 167 (16.2%) had one or more ADRs; 15 (7.5%) of the 199 ADRs were classified as severe [20]. Sunshine et al. found that surgical and perioperative events accounted for 63.6% of all deaths due to AEMT in the United States [16]. Schwendimann et al. [21] conducted a scoping review of retrospective medical record studies, 25 studies conducted in 27 countries across six continents, to determine the proportions of patients affected by in-hospital adverse events, explore their types and consequences, and estimate the preventability of in-hospital adverse events. They found that a median of 10% of patients were affected by at least one adverse event (range, 2.9%–21.9%), with a median of 7.3% (range, 0.6%–30%) being fatal. Almost half of the adverse events were considered preventable. The three most common types of adverse events reported were surgery-related, medication- or drug/fluid-related, and healthcare-associated infections. An indication of the extent of underreporting in India is indirectly provided by the statistics on ADR reporting. India employs voluntary/spontaneous mechanisms for ADR reporting, and under-reporting of ADRs is pervasive and challenging to combat [22]. Under-reporting can be caused by patient-related factors such as a patient's incapacity to identify an ADR or to associate the ADR with a medication. Physician-related causes include feelings of guilt, lawsuit fear, ignorance, laziness, inadequate awareness of risks concerning recently marketed pharmaceuticals, hesitation, insufficient training to recognize ADR, and ignorance of the national program [23]. ADRs may go unreported because of the inability of a medical team to accurately correlate ADRs with biochemical, pathological, or radiological abnormalities [24]. The ADR reporting rates of various ADR monitoring centers are highly variable, with a small percentage of such centers contributing to the majority of the reported incidents [22]. Given that the challenges in reporting such events are, to some extent, common throughout the world, it is important to determine measures that can encourage the reporting culture. In fact, the resolution on ‘Global action on patient safety’ adopted by the World Health Organization in 2019 emphasizes the importance of tackling healthcare-related patient harm despite the apparent absence of robust data [25].

A review of systematic reviews identified decision-making errors, such as not considering a patient risk factor, as the most common cause of medication errors, followed by organizational factors (lack of staff) and environmental factors (distractions) [26]. Several measures have been studied to decrease adverse medical events, although not all have been successful, and almost none have equivocally proved beneficial [27,28]. Technology-enabled systems can be useful in avoiding or decreasing medication mistakes. Automated dispensing devices, pharmacy-based computer systems, computerized physician order entry, and renal dosing alerts are a few examples [29]. Beers criteria is a widely used tool to prevent/correct the prescription of potentially inappropriate medications, and thereby, ensure treatment safety in elderly patients [30]. AI has been used to automate the processes for filing adverse event reports during the drug development phase. In a study conducted in Spain, healthcare quality professionals examined the capabilities of AI to see how effectively it may have prevented the event detailed in the clinical case. AI was determined to be able to significantly prevent adverse incidents [31]. The Information Systems-enabled Outreach for Preventing Adverse Drug Events (ISTOP-ADE system) was assessed in a Canadian study of 568 patients; this involves an interactive voice response system that calls patients to see if they have any issues and to put them in touch with a chemist if that is what the patient needs or wants. The program found 10 of 26 primary non-compliant events and 56 of 125 (45%) ADEs. This intervention was determined to enhance quality patient care by reducing the duration of ADE and enhancing compliance with medications [32]. A study conducted in France in an intensive care unit setting of a tertiary university medical facility used a multidisciplinary committee to examine each event report using the standardized Orion methodology. Recognition, timeline, root-cause evaluation, and reviewing standard-of-care protocols were all included in the analysis report. The committee also decided on the ultimate corrective measures. However, the low number of incidents in each category and the considerable staff turnover in the intensive care unit prevented this method from being successful [33].

Given the presence of gross underreporting, incomplete coverage of medical certification of deaths, and hospital-based studies reporting a higher percentage of hospitalizations and deaths being treatment-related, it is important to strengthen the identification and reporting structure in healthcare institutions. Importantly, there is a need to comprehensively review available evidence-based safeguards to prevent AEMT which can be cost-effectively implemented in India. Implementation of strong policies, reframing of service delivery to patients, enforcement of appropriate patient safety training to healthcare workers, implementation of individual liability for quality and patient safety by clearer and transparent documentation, and development of a safe culture of feedback are required to achieve the World Health Organization patient safety strategy goals.

In interpreting the results of our study, it is important to consider the limitations of the GBD modeling process and to understand the potential deficiencies in the MCCD data. The estimates generated using the GBD modeling process are based on the source data; scanty or poor-quality source data leads to estimates with large uncertainty intervals. This is true of the AEMT data for India; the underlying data for generating the estimates was based on MCCD reports from 2009 to 2012, Odisha MCCD reports from 2009 to 2013, Karnataka MCCD reports from 2014 to 2015, and Delhi MCCD report for 2013. The modeling process uses these data and generates estimates for other regions of the country. Hence, despite many states not reporting any deaths due to AEMT, the GBD process nonetheless generates estimates based on population size and other factors. This probably explains why the numbers estimated using the GBD modeling process are much higher than those reported in the MCCD reports. As described earlier, the assumption is that a crude estimate is better than no estimate for a cause/disease because the former helps in health planning and resource allocation. In this context, an interesting aspect to consider is the AEMT deaths in the United States as estimated by GBD and the numbers reported by the US DHHS. As per the GBD data, the number of deaths in the US in 2019 was 5016. However, a study conducted by DHHS in 2018 in a representative sample of 770 patients from 1076344 Medicare users estimated that of the 1 million population, 14800 patients died due to adverse events, and 121089 experienced serious adverse events [5]. Hence, despite a good underlying data source for the US GBD estimate, it seems to be much lower than the actual number of deaths.

Regarding MCCD, because the scheme is being implemented in a phased manner and only selected hospitals from each state are currently included, the number of deaths reported is much less than the actual numbers. In 2019, the total medically certified deaths due to all causes accounted for 20.7% of the total certified deaths [13]. Regarding AEMT deaths, of the 35 states and union territories, one state accounted for 83% of the reported deaths and 18 states/union territories reported no cases; in 2010, three states accounted for 81% of the total reported deaths with 18 states/union territories reporting no cases [13]. The disparity between the reported numbers from various states suggests that either deaths due to AEMT are probably being coded to other causes of death or there is an underdiagnosis of drugs or treatments as having been responsible for the deaths. Given the sensitivities and challenges involved in reporting harm/death due to treatment, significant under-reporting is to be expected. Another obvious cause for discrepancy between the GBD and MCCD data is that the former includes ICD-10 code Y88–Y88.3 ‘sequelae with surgical and medical care as external cause’ under AEMT, whereas in MCCD, this forms part of ‘Late effects of external causes of morbidity and mortality’ (Y85–Y89) and is not included in AEMT. However, this only partly explains the disparity given that even if all cases reported under Y85–Y89 were to be added to deaths due to AEMT, it would account for only a small percentage of the discrepancy.

## 5 Conclusion

To conclude, India has seen an increase in the incidence rate of AEMT but a decrease in death and DALY rates over the past decade. The disease burden is more in females and those aged ≥75 years. AEMT accounts for only a small percentage of deaths due to all causes; however, the impact of medical treatment-related deaths on the public perception regarding healthcare services as well as the potential underreporting of AEMT cases need to be studied.

## Ethics statement

The study protocol was approved by the 10.13039/100019519Kasturba Medical College Institutional Ethics Committee (IEC KMCMLR-12/2022/497). Informed consent was not required for this study because the study used data from databases accessible to the public.

## Sources of funding

This research did not receive any specific grant from funding agencies in the public, commercial, or not-for-profit sectors.

## Data availability statement

The data associated with this article are presented in the manuscript and supplementary material. The data were obtained from the Global Burden of Disease 2019 database, which can be accessed at https://vizhub.healthdata.org/gbd-results/.

## CRediT authorship contribution statement

**Ashwin Kamath:** Writing – review & editing, Visualization, Methodology, Formal analysis, Data curation, Conceptualization. **Sahana D. Acharya:** Writing – original draft, Methodology, Conceptualization. **Poovizhi Bharathi R:** Writing – original draft, Visualization, Methodology, Formal analysis.

## Declaration of competing interest

The authors declare the following financial interests/personal relationships which may be considered as potential competing interests:The corresponding author is an advisory board member of Heliyon.
