# Supplementary material for: Burden of death and disability due to adverse effects of medical treatment in India: An analysis using the global burden of disease 2019 study data
Source: Heliyon. 2024 Jan 18;10(2):e24924. doi: 10.1016/j.heliyon.2024.e24924 (PMC10835318; doi:10.1016/j.heliyon.2024.e24924)
Supplement: Multimedia component 1 [file mmc1.docx]

Supplementary Table 1. International Classification of Diseases version 10 codes for adverse effects of medical treatment used in the global burden of disease 2019 study

| **ICD-10 code*** | **Category** |
| --- | --- |
| N30.4 | Irradiation cystitis |
| Y40-Y59 | Drugs, medicaments and biological substances causing adverse effects in therapeutic use |
| Y60-Y69 | Misadventures to patients during surgical and medical care |
| Y70-Y82 | Medical devices associated with adverse incidents in diagnostic and therapeutic use |
| Y83-84 | Surgical and other medical procedures as the cause of abnormal reaction of the patient, or of later complication, without mention of misadventure at the time of the procedure |
| Y88 | Sequelae with surgical and medical care as external cause |

*The codes include codes for subcategories unless the latter has been specified.

Supplementary Table 2. Country-level age-wise distribution of death and DALY numbers and rates due to adverse effects of medical treatment in the year 2019

| **Variable** | **Year** | **Age group (in years)** | | | | |
| --- | --- | --- | --- | --- | --- | --- |
|  |  | **0 to 9** | **10 to 24** | **25 to 49** | **50 to 74** | **75 plus** |
| **Death** |  |  |  |  |  |  |
| Percentage | 2010 | 8.71 | 6.76 | 19.20 | 45.07 | 20.26 |
|  | 2019 | 4.42 | 4.69 | 16.76 | 45.83 | 28.31 |
| Number, N (95% UI) | 2010 | 2512 (1714–3191) | 1949 (1450–2251) | 5534 (4076–6324) | 12995 (9504–14928) | 5840 (4317–6910) |
|  | 2019 | 1435 (980–1920) | 1521(1138–1861) | 5440 (3871–6874) | 14877 (10684–18666) | 9191 (6762–11476) |
| Rate | 2010 | 0.98 (0.67–1.24) | 0.52 (0.39–0.61) | 1.34 (0.98–1.53) | 7.71 (5.64–8.86) | 29.27 (21.63–34.63) |
|  | 2019 | 0.59 (0.40–0.79) | 0.38 (0.29–0.47) | 1.10 (0.78–1.39) | 6.64 (4.77–8.33) | 29.71 (21.86–37.10) |
| **DALY** |  |  |  |  |  |  |
| Percentage | 2010 | 21.11 | 13.10 | 27.02 | 31.75 | 7.02 |
|  | 2019 | 12.89 | 10.70 | 27.51 | 38.09 | 10.82 |
| Number, N (95% UI) | 2010 | 223393 (152006–283101) | 138652 (103413–159696) | 285988 (212980–325167) | 336037 (245959–385472) | 74304 (55256–87570) |
|  | 2019 | 131489 (92254–173904) | 109144 (82562–132370) | 280777 (202770–352877) | 388668 (278699–490352) | 110374 (81036–138197) |
| Rate | 2010 | 41.69 (28.37–42.93) | 37.27 (27.80–52.84) | 69.08 (51.45–78.55) | 105.80 (77.44–121.36) | 265.95 (197.78–313.44) |
|  | 2019 | 26.31 (18.46–34.80) | 27.47 (20.78–33.31) | 56.64 (40.90–71.18) | 92.00 (65.97–116.06) | 243.10 (178.48–304.38) |

UI, uncertainty interval; DALY, disability adjusted life year

Supplementary Table 3. Country-level gender-wise distribution of death and DALY numbers and rates due to adverse effects of medical treatment in the year 2010 and 2019

| **Measure** | **2019** | | | | **2010** | | | |
| --- | --- | --- | --- | --- | --- | --- | --- | --- |
|  | **Male** | **95% UI** | **Female** | **95% UI** | **Male** | **95% UI** | **Female** | **95% UI** |
| Death number | 13704 | 9260–17771 | 18760 | 12244–24263 | 12969 | 8997–15161 | 15862 | 11051–18625 |
| Death rate | 1.92 | 1.30–2.49 | 2.77 | 1.81–3.58 | 2.04 | 1.42–2.40 | 2.66 | 1.85–3.12 |
| DALY number | 408139 | 282433–517872 | 612314 | 406869–774798 | 446735 | 312702–518344 | 611640 | 427125–707977 |
| DALY rate | 57.23 | 39.60–72.61 | 90.38 | 60.05–114.36 | 70.44 | 49.31–81.73 | 102.42 | 71.53–118.56 |

UI, uncertainty interval; DALY, disability adjusted life year

Supplementary Table 4. Country-wise death and DALY rates due to adverse effects of medical treatment in the year 2010 and 2019

| **Country / Territories** | **Death rate 2010** | **95% UI** | **Death rate 2019** | **95% UI** | **DALY rate 2010** | **95% UI** | **DALY rate 2019** | **95% UI** |
| --- | --- | --- | --- | --- | --- | --- | --- | --- |
| Afghanistan | 3.42 | 1.65–5.55 | 2.52 | 1.29–3.96 | 193.91 | 98.15–300.43 | 140.41 | 73.76–214.76 |
| Albania | 0.85 | 0.63–1.01 | 0.96 | 0.71–1.25 | 37.34 | 24.50–47.53 | 35.96 | 24.47–49.41 |
| Algeria | 1.13 | 0.64–1.53 | 1.02 | 0.59–1.36 | 52.15 | 29.67–69.69 | 42.12 | 24.80–56.15 |
| American Samoa | 2.13 | 1.79–2.72 | 2.66 | 2.05–3.34 | 84.06 | 69.87–106.71 | 98.09 | 72.59–129.21 |
| Andorra | 0.48 | 0.38–0.62 | 0.56 | 0.42–0.73 | 12.58 | 9.96–15.76 | 13.28 | 9.94–17.14 |
| Angola | 2.30 | 1.56–3.08 | 1.35 | 0.94–1.82 | 151.52 | 96.31–219.29 | 77.62 | 53.65–108.35 |
| Antigua and Barbuda | 1.80 | 1.53–2.01 | 2.05 | 1.68–2.41 | 50.39 | 44.48–56.80 | 54.27 | 44.36–65.21 |
| Argentina | 4.03 | 3.58–4.50 | 3.92 | 3.45–4.38 | 98.12 | 90.51–111.88 | 93.48 | 84.92–106.66 |
| Armenia | 0.45 | 0.40–0.50 | 0.53 | 0.39–0.63 | 17.89 | 15.86–20.21 | 18.70 | 15.16–21.84 |
| Australia | 1.57 | 1.08–1.74 | 1.53 | 1.15–1.72 | 42.73 | 34.71–49.96 | 41.38 | 34.37–49.13 |
| Austria | 2.10 | 1.72–2.29 | 2.41 | 1.89–2.67 | 48.28 | 36.72–52.26 | 53.27 | 38.64–59.33 |
| Azerbaijan | 0.26 | 0.21–0.31 | 0.23 | 0.18–0.28 | 13.55 | 10.62–16.01 | 11.44 | 8.99–13.87 |
| Bahamas | 2.07 | 1.84–2.31 | 2.42 | 1.93–2.98 | 71.23 | 61.86–78.79 | 76.32 | 60.62–96.45 |
| Bahrain | 0.40 | 0.34–0.55 | 0.45 | 0.35–0.62 | 20.76 | 17.60–26.06 | 20.08 | 16.22–25.96 |
| Bangladesh | 1.97 | 1.43–2.97 | 1.90 | 1.33–2.74 | 75.43 | 55.52–112.42 | 59.09 | 41.95–87.26 |
| Barbados | 3.50 | 3.00–3.92 | 3.93 | 3.24–4.76 | 82.83 | 72.55–92.54 | 87.17 | 70.44–107.86 |
| Belarus | 0.58 | 0.54–0.71 | 0.55 | 0.42–0.73 | 21.28 | 19.33–24.08 | 19.09 | 14.71–24.90 |
| Belgium | 2.60 | 2.18–2.85 | 2.58 | 2.17–2.91 | 45.07 | 40.15–51.95 | 42.80 | 37.87–51.44 |
| Belize | 3.17 | 2.83–3.49 | 3.44 | 2.94–4.03 | 126.26 | 110.98–138.68 | 127.77 | 107.21–150.44 |
| Benin | 2.77 | 1.90–3.91 | 2.11 | 1.44–2.97 | 180.68 | 112.33–274.03 | 131.42 | 85.63–194.15 |
| Bermuda | 1.34 | 1.15–1.52 | 1.71 | 1.39–2.06 | 29.95 | 26.43–35.11 | 33.36 | 27.67–40.46 |
| Bhutan | 2.29 | 1.54–3.74 | 2.31 | 1.58–3.61 | 80.92 | 53.32–133.05 | 68.74 | 44.56–112.45 |
| Bolivia (Plurinational State of) | 1.35 | 1.05–1.76 | 1.22 | 0.92–1.60 | 62.49 | 47.05–81.77 | 49.08 | 36.29–66.66 |
| Bosnia and Herzegovina | 1.28 | 0.60–3.08 | 1.42 | 0.57–3.63 | 39.61 | 22.50–82.24 | 38.76 | 18.90–87.43 |
| Botswana | 1.42 | 0.94–1.96 | 1.33 | 0.89–1.90 | 69.59 | 46.41–95.65 | 62.21 | 41.82–88.26 |
| Brazil | 1.21 | 1.03–1.28 | 1.36 | 1.09–1.46 | 41.32 | 36.30–44.86 | 40.61 | 33.79–44.24 |
| Brunei Darussalam | 0.47 | 0.38–0.54 | 0.55 | 0.43–0.66 | 20.73 | 16.34–23.67 | 22.22 | 16.98–26.77 |
| Bulgaria | 0.92 | 0.84–1.13 | 0.98 | 0.79–1.25 | 30.44 | 27.10–33.51 | 30.12 | 24.17–37.46 |
| Burkina Faso | 3.15 | 1.98–4.43 | 3.12 | 2.09–4.36 | 203.86 | 119.14–304.97 | 203.53 | 128.48–300.92 |
| Burundi | 2.57 | 1.52–4.98 | 1.98 | 1.11–4.25 | 158.51 | 90.02–263.84 | 112.43 | 64.81–216.55 |
| CÃ´te d'Ivoire | 1.98 | 1.32–2.71 | 1.57 | 1.06–2.12 | 117.56 | 74.39–170.87 | 87.17 | 56.67–123.45 |
| Cabo Verde | 0.96 | 0.73–1.14 | 1.05 | 0.74–1.32 | 39.06 | 30.31–47.79 | 34.42 | 25.19–44.25 |
| Cambodia | 0.99 | 0.67–1.55 | 0.90 | 0.62–1.46 | 46.12 | 32.07–73.16 | 36.68 | 25.49–57.76 |
| Cameroon | 2.07 | 1.36–2.81 | 1.57 | 0.99–2.25 | 121.22 | 73.69–176.69 | 86.46 | 51.90–128.19 |
| Canada | 0.99 | 0.88–1.16 | 1.08 | 0.94–1.28 | 30.58 | 26.04–37.49 | 31.87 | 26.63–39.39 |
| Central African Republic | 3.27 | 2.11–4.52 | 2.65 | 1.76–3.80 | 204.58 | 111.39–306.29 | 152.67 | 95.54–221.59 |
| Chad | 3.47 | 2.45–4.68 | 2.69 | 1.93–3.68 | 223.04 | 153.28–325.01 | 172.55 | 120.47–252.24 |
| Chile | 0.94 | 0.83–1.28 | 1.06 | 0.93–1.39 | 25.63 | 22.97–34.50 | 27.41 | 24.29–36.14 |
| China | 0.38 | 0.33–0.50 | 0.34 | 0.28–0.45 | 15.18 | 13.44–19.40 | 11.67 | 9.69–15.16 |
| Colombia | 0.54 | 0.48–0.67 | 0.65 | 0.50–0.84 | 17.58 | 15.97–22.89 | 19.00 | 14.60–24.71 |
| Comoros | 1.75 | 1.13–2.97 | 1.69 | 1.05–3.08 | 81.95 | 53.34–121.47 | 69.94 | 43.89–114.49 |
| Congo | 1.52 | 1.05–1.96 | 1.17 | 0.79–1.57 | 78.02 | 50.17–105.03 | 52.00 | 34.96–71.09 |
| Cook Islands | 0.25 | 0.19–0.29 | 0.29 | 0.20–0.37 | 8.40 | 6.81–9.91 | 8.65 | 6.24–11.10 |
| Costa Rica | 1.75 | 1.52–1.98 | 2.05 | 1.57–2.64 | 51.56 | 47.14–62.54 | 55.90 | 43.32–72.39 |
| Croatia | 1.30 | 0.99–1.44 | 1.30 | 0.95–1.64 | 35.88 | 28.18–39.79 | 33.41 | 25.03–42.08 |
| Cuba | 0.79 | 0.70–1.16 | 0.88 | 0.70–1.26 | 21.14 | 18.85–29.53 | 21.94 | 17.61–29.88 |
| Cyprus | 1.51 | 1.04–1.77 | 1.56 | 1.04–1.92 | 31.71 | 23.84–36.12 | 31.43 | 22.70–37.74 |
| Czechia | 1.31 | 0.63–1.50 | 2.00 | 0.72–2.61 | 32.19 | 19.78–36.71 | 43.88 | 21.91–56.16 |
| Democratic People's Republic of Korea | 0.68 | 0.50–0.85 | 0.60 | 0.45–0.79 | 29.19 | 22.07–37.20 | 22.62 | 16.54–29.97 |
| Democratic Republic of the Congo | 2.23 | 1.46–3.79 | 1.47 | 0.98–2.44 | 128.91 | 80.27–189.30 | 75.26 | 51.25–111.93 |
| Denmark | 1.33 | 0.76–1.51 | 1.54 | 0.83–1.79 | 26.28 | 16.63–29.23 | 29.12 | 17.79–33.32 |
| Djibouti | 1.92 | 1.22–2.98 | 1.47 | 0.94–2.59 | 117.88 | 76.63–169.72 | 78.27 | 51.04–121.54 |
| Dominica | 3.72 | 3.04–4.56 | 3.85 | 3.01–4.93 | 95.63 | 77.77–115.78 | 97.86 | 74.75–127.53 |
| Dominican Republic | 2.49 | 1.55–3.31 | 2.23 | 1.50–3.22 | 87.37 | 56.56–114.79 | 75.98 | 50.13–107.89 |
| Ecuador | 0.69 | 0.59–0.93 | 0.65 | 0.51–0.87 | 28.72 | 23.94–39.07 | 23.93 | 18.87–31.72 |
| Egypt | 0.71 | 0.46–0.87 | 0.60 | 0.37–0.87 | 32.15 | 22.02–39.45 | 25.64 | 16.81–35.22 |
| El Salvador | 0.48 | 0.38–0.70 | 0.52 | 0.36–0.79 | 16.40 | 13.19–23.92 | 15.67 | 10.86–24.04 |
| Equatorial Guinea | 0.96 | 0.59–1.39 | 0.65 | 0.41–1.01 | 54.77 | 31.71–83.01 | 31.62 | 19.01–49.78 |
| Eritrea | 2.04 | 1.33–3.31 | 1.74 | 1.09–3.02 | 118.56 | 77.60–171.33 | 92.22 | 59.26–148.15 |
| Estonia | 0.98 | 0.74–1.09 | 0.92 | 0.68–1.17 | 25.47 | 21.02–27.99 | 22.31 | 17.10–27.88 |
| Eswatini | 1.76 | 1.19–2.44 | 1.44 | 0.98–1.97 | 96.85 | 64.08–136.98 | 73.32 | 49.64–101.41 |
| Ethiopia | 2.30 | 1.25–5.09 | 1.60 | 0.88–3.67 | 131.50 | 79.62–242.98 | 82.72 | 49.76–167.95 |
| Fiji | 1.00 | 0.86–1.19 | 0.99 | 0.75–1.29 | 44.88 | 38.09–52.50 | 42.03 | 31.82–54.36 |
| Finland | 0.50 | 0.43–0.67 | 0.58 | 0.50–0.75 | 12.65 | 11.14–15.19 | 13.17 | 11.47–15.40 |
| France | 3.60 | 3.09–4.14 | 3.66 | 3.04–4.33 | 59.85 | 53.65–75.33 | 56.81 | 48.97–74.66 |
| Gabon | 1.37 | 0.97–1.76 | 1.08 | 0.73–1.43 | 62.56 | 42.80–83.53 | 43.21 | 29.25–58.05 |
| Gambia | 1.90 | 1.37–2.76 | 1.61 | 1.14–2.28 | 101.14 | 70.27–148.07 | 76.00 | 51.24–114.46 |
| Georgia | 1.14 | 0.73–1.29 | 1.33 | 0.72–1.60 | 30.99 | 22.86–34.58 | 35.56 | 22.95–42.14 |
| Germany | 2.16 | 1.55–2.34 | 2.56 | 1.73–2.88 | 45.09 | 31.84–48.58 | 50.55 | 34.34–56.02 |
| Ghana | 2.06 | 1.53–2.56 | 1.74 | 1.26–2.25 | 105.42 | 75.01–137.23 | 83.65 | 58.75–113.27 |
| Greece | 2.17 | 1.91–2.36 | 3.53 | 2.67–3.93 | 48.78 | 42.24–52.39 | 70.77 | 49.66–78.80 |
| Greenland | 0.78 | 0.60–0.92 | 0.83 | 0.60–1.04 | 31.38 | 25.76–36.96 | 31.07 | 24.50–38.44 |
| Grenada | 3.44 | 3.10–3.78 | 3.37 | 2.97–3.86 | 108.29 | 97.43–120.78 | 99.14 | 85.60–115.86 |
| Guam | 0.72 | 0.60–0.86 | 1.03 | 0.70–1.33 | 28.64 | 23.89–33.33 | 36.11 | 24.29–47.06 |
| Guatemala | 1.08 | 0.89–1.73 | 1.01 | 0.73–1.83 | 47.53 | 39.76–72.52 | 40.52 | 29.33–71.58 |
| Guinea | 3.45 | 2.45–4.62 | 2.72 | 1.85–3.70 | 203.50 | 134.08–294.24 | 161.28 | 105.82–233.75 |
| Guinea-Bissau | 3.05 | 2.25–3.95 | 2.12 | 1.55–2.78 | 180.93 | 130.67–247.65 | 111.51 | 80.43–150.87 |
| Guyana | 2.67 | 2.32–3.04 | 2.87 | 2.20–3.68 | 104.02 | 89.20–118.55 | 107.70 | 81.05–142.26 |
| Haiti | 3.81 | 2.85–5.02 | 3.50 | 2.56–4.69 | 198.02 | 141.01–273.67 | 173.13 | 123.23–235.73 |
| Honduras | 1.90 | 0.81–3.04 | 1.85 | 0.76–3.09 | 79.35 | 31.85–124.09 | 67.39 | 26.07–112.57 |
| Hungary | 1.01 | 0.69–1.13 | 1.04 | 0.72–1.31 | 25.29 | 19.15–28.12 | 25.19 | 18.80–31.00 |
| Iceland | 0.98 | 0.84–1.14 | 1.22 | 0.96–1.41 | 20.64 | 18.37–24.42 | 23.84 | 20.13–27.31 |
| India | 2.34 | 1.75–2.66 | 2.33 | 1.73–2.86 | 85.95 | 64.32–97.00 | 73.38 | 54.74–88.49 |
| Indonesia | 0.67 | 0.41–1.11 | 0.60 | 0.34–1.03 | 28.79 | 17.03–46.98 | 22.72 | 12.74–38.41 |
| Iran (Islamic Republic of) | 1.27 | 1.03–1.43 | 1.04 | 0.90–1.29 | 58.63 | 46.34–64.01 | 39.55 | 34.45–46.78 |
| Iraq | 0.69 | 0.54–0.87 | 0.55 | 0.41–0.72 | 37.84 | 29.08–46.90 | 27.54 | 20.55–35.66 |
| Ireland | 0.85 | 0.67–0.94 | 0.77 | 0.65–0.87 | 20.92 | 16.93–22.95 | 17.61 | 15.18–19.70 |
| Israel | 2.00 | 1.65–3.39 | 2.06 | 1.65–3.46 | 46.56 | 39.73–75.73 | 45.20 | 37.76–75.46 |
| Italy | 1.38 | 1.11–1.50 | 1.62 | 1.26–1.78 | 23.57 | 20.67–26.18 | 25.63 | 21.48–27.75 |
| Jamaica | 2.27 | 1.47–2.54 | 2.53 | 1.59–3.21 | 60.55 | 39.05–67.68 | 66.18 | 40.20–86.14 |
| Japan | 1.08 | 0.88–1.18 | 1.33 | 1.02–1.49 | 22.91 | 18.43–24.36 | 24.30 | 19.61–26.34 |
| Jordan | 0.30 | 0.25–0.36 | 0.28 | 0.23–0.36 | 17.49 | 14.51–20.83 | 15.62 | 12.53–19.11 |
| Kazakhstan | 0.61 | 0.56–0.73 | 0.65 | 0.52–0.77 | 26.78 | 24.09–31.77 | 27.01 | 21.83–31.88 |
| Kenya | 1.24 | 0.84–2.26 | 1.04 | 0.69–1.85 | 63.72 | 46.65–103.79 | 48.23 | 33.71–81.89 |
| Kiribati | 3.31 | 2.21–5.27 | 3.07 | 2.05–4.89 | 158.91 | 111.37–248.24 | 141.29 | 97.13–218.55 |
| Kuwait | 0.59 | 0.53–0.70 | 0.76 | 0.62–0.91 | 25.70 | 23.15–30.08 | 30.60 | 24.93–36.96 |
| Kyrgyzstan | 0.24 | 0.20–0.26 | 0.23 | 0.18–0.27 | 11.68 | 10.06–13.27 | 11.52 | 9.53–13.55 |
| Lao People's Democratic Republic | 1.02 | 0.70–1.63 | 0.79 | 0.51–1.32 | 55.41 | 34.74–88.94 | 37.31 | 23.87–62.04 |
| Latvia | 1.05 | 0.69–1.19 | 1.20 | 0.71–1.51 | 27.88 | 20.17–30.96 | 30.10 | 19.91–37.32 |
| Lebanon | 1.15 | 0.70–1.51 | 0.98 | 0.57–1.35 | 40.34 | 24.10–52.66 | 34.14 | 19.95–47.42 |
| Lesotho | 2.14 | 1.56–2.78 | 1.93 | 1.33–2.52 | 98.25 | 71.59–124.93 | 87.08 | 60.43–112.84 |
| Liberia | 1.95 | 1.35–2.77 | 1.48 | 1.01–2.12 | 107.37 | 72.73–152.73 | 74.14 | 49.76–107.72 |
| Libya | 0.94 | 0.55–1.28 | 1.03 | 0.58–1.52 | 43.30 | 24.64–60.38 | 41.42 | 23.53–61.29 |
| Lithuania | 0.99 | 0.74–1.08 | 1.22 | 0.78–1.53 | 28.10 | 22.18–30.67 | 32.47 | 22.07–40.25 |
| Luxembourg | 2.14 | 1.88–2.50 | 2.23 | 1.85–2.64 | 42.34 | 38.19–50.44 | 43.19 | 36.71–52.38 |
| Madagascar | 1.64 | 1.09–2.68 | 1.26 | 0.83–2.13 | 96.65 | 66.24–142.24 | 68.04 | 47.11–102.17 |
| Malawi | 2.15 | 1.36–3.94 | 1.58 | 0.95–3.07 | 127.74 | 85.22–196.39 | 85.32 | 54.59–145.40 |
| Malaysia | 0.63 | 0.50–0.72 | 0.66 | 0.49–0.86 | 22.63 | 18.61–25.54 | 21.76 | 16.44–28.06 |
| Maldives | 1.01 | 0.86–1.27 | 0.93 | 0.75–1.13 | 46.36 | 38.63–57.06 | 37.22 | 29.45–45.38 |
| Mali | 3.53 | 2.35–5.02 | 3.06 | 2.05–4.28 | 229.73 | 141.41–339.91 | 200.27 | 126.33–292.12 |
| Malta | 1.13 | 0.98–1.28 | 1.28 | 1.06–1.49 | 24.64 | 22.02–28.25 | 25.64 | 21.99–30.00 |
| Marshall Islands | 2.13 | 1.52–3.20 | 2.02 | 1.38–3.18 | 105.45 | 75.92–156.17 | 92.85 | 64.60–144.79 |
| Mauritania | 1.44 | 1.11–1.86 | 1.11 | 0.76–1.53 | 68.90 | 52.51–89.48 | 46.77 | 30.20–68.47 |
| Mauritius | 2.98 | 2.09–3.32 | 4.09 | 2.31–5.27 | 104.23 | 72.36–116.02 | 126.51 | 73.43–164.56 |
| Mexico | 0.89 | 0.78–0.98 | 0.97 | 0.80–1.13 | 33.33 | 29.62–37.32 | 32.01 | 27.32–36.90 |
| Micronesia (Federated States of) | 1.96 | 1.40–2.98 | 2.00 | 1.36–3.14 | 86.55 | 62.81–130.53 | 84.99 | 55.96–133.19 |
| Monaco | 14.67 | 11.68–17.72 | 13.51 | 10.48–16.46 | 250.64 | 200.05–302.83 | 229.31 | 177.30–283.88 |
| Mongolia | 0.31 | 0.24–0.39 | 0.28 | 0.20–0.37 | 17.18 | 13.66–20.67 | 14.54 | 11.12–18.58 |
| Montenegro | 0.47 | 0.39–0.54 | 0.45 | 0.36–0.56 | 15.46 | 12.91–17.69 | 14.23 | 11.66–17.21 |
| Morocco | 1.71 | 0.87–2.62 | 1.51 | 0.83–2.16 | 73.92 | 40.17–107.89 | 55.81 | 31.77–81.09 |
| Mozambique | 2.94 | 1.73–5.53 | 2.20 | 1.28–4.25 | 178.43 | 110.02–287.59 | 126.30 | 76.96–216.45 |
| Myanmar | 1.07 | 0.68–1.79 | 0.86 | 0.56–1.49 | 52.43 | 29.64–89.25 | 36.96 | 22.14–65.20 |
| Namibia | 1.32 | 0.98–1.73 | 1.19 | 0.83–1.63 | 61.39 | 44.18–82.51 | 51.70 | 35.03–73.41 |
| Nauru | 2.08 | 1.30–3.16 | 1.53 | 0.92–2.34 | 115.84 | 69.01–178.42 | 82.25 | 49.74–125.12 |
| Nepal | 2.14 | 1.55–3.25 | 2.31 | 1.65–3.33 | 75.83 | 54.01–114.88 | 70.13 | 49.93–102.86 |
| Netherlands | 0.72 | 0.64–0.90 | 0.79 | 0.68–1.01 | 15.35 | 13.84–19.43 | 15.70 | 13.86–20.18 |
| New Zealand | 0.55 | 0.48–0.77 | 0.69 | 0.60–0.88 | 30.83 | 23.96–39.45 | 34.12 | 26.63–43.55 |
| Nicaragua | 0.66 | 0.56–0.88 | 0.66 | 0.51–0.92 | 24.27 | 20.80–31.43 | 22.08 | 17.40–29.93 |
| Niger | 3.41 | 2.10–5.37 | 3.01 | 1.88–4.70 | 229.56 | 137.47–351.56 | 199.12 | 122.79–312.27 |
| Nigeria | 2.37 | 1.57–3.18 | 1.74 | 1.23–2.34 | 156.85 | 95.64–220.96 | 110.77 | 71.70–155.24 |
| Niue | 1.96 | 1.33–2.93 | 1.75 | 1.16–2.64 | 59.22 | 39.24–89.52 | 52.22 | 34.08–80.52 |
| North Macedonia | 0.40 | 0.34–0.47 | 0.40 | 0.31–0.51 | 13.43 | 11.50–15.47 | 13.10 | 10.47–16.14 |
| Northern Mariana Islands | 1.23 | 0.98–1.53 | 1.92 | 1.30–2.44 | 49.60 | 38.87–62.76 | 64.25 | 43.09–84.37 |
| Norway | 0.54 | 0.47–0.61 | 0.53 | 0.45–0.60 | 13.85 | 11.99–16.38 | 12.61 | 10.89–14.91 |
| Oman | 0.75 | 0.61–0.86 | 0.48 | 0.38–0.58 | 35.92 | 29.02–41.35 | 24.02 | 19.12–29.38 |
| Pakistan | 2.34 | 1.88–2.85 | 2.12 | 1.71–2.62 | 104.02 | 81.60–128.00 | 91.19 | 70.58–113.98 |
| Palau | 12.52 | 9.54–16.08 | 13.02 | 9.74–16.92 | 498.40 | 370.24–644.29 | 476.43 | 348.85–622.41 |
| Palestine | 0.45 | 0.37–0.61 | 0.43 | 0.35–0.58 | 24.23 | 19.55–31.01 | 20.58 | 16.98–26.03 |
| Panama | 0.44 | 0.36–0.80 | 0.46 | 0.34–0.83 | 15.77 | 13.20–27.01 | 15.94 | 11.61–27.18 |
| Papua New Guinea | 2.10 | 1.25–3.37 | 1.90 | 1.13–3.04 | 117.24 | 75.56–180.52 | 102.96 | 65.08–160.26 |
| Paraguay | 1.54 | 1.13–1.80 | 1.66 | 1.04–2.28 | 52.74 | 40.04–61.78 | 51.25 | 33.40–70.95 |
| Peru | 0.96 | 0.78–1.24 | 0.82 | 0.58–1.16 | 40.02 | 33.32–49.18 | 30.35 | 21.87–43.13 |
| Philippines | 0.45 | 0.38–0.54 | 0.44 | 0.34–0.53 | 22.31 | 18.65–26.32 | 20.21 | 15.56–24.37 |
| Poland | 1.37 | 0.93–1.48 | 1.46 | 0.98–1.75 | 36.90 | 27.60–40.23 | 36.25 | 26.31–43.44 |
| Portugal | 2.28 | 2.05–2.72 | 2.58 | 2.24–3.10 | 50.28 | 45.88–55.67 | 51.00 | 44.73–57.29 |
| Puerto Rico | 2.12 | 1.78–2.34 | 2.97 | 2.17–3.80 | 44.27 | 40.07–49.12 | 54.90 | 42.08–70.17 |
| Qatar | 0.34 | 0.24–0.44 | 0.29 | 0.18–0.40 | 17.62 | 13.88–21.45 | 14.61 | 10.77–18.61 |
| Republic of Korea | 0.35 | 0.25–0.40 | 0.40 | 0.27–0.48 | 10.18 | 7.38–11.46 | 9.63 | 6.62–11.33 |
| Republic of Moldova | 0.48 | 0.44–0.62 | 0.48 | 0.41–0.62 | 17.20 | 15.39–20.32 | 15.57 | 13.08–19.17 |
| Romania | 0.53 | 0.49–0.65 | 0.55 | 0.46–0.72 | 16.73 | 15.14–19.06 | 16.11 | 13.20–20.07 |
| Russian Federation | 1.12 | 0.86–1.17 | 1.12 | 0.77–1.32 | 38.63 | 30.33–41.02 | 35.38 | 25.83–41.29 |
| Rwanda | 1.87 | 1.25–3.00 | 1.46 | 0.95–2.61 | 112.29 | 77.23–157.58 | 75.07 | 49.36–121.33 |
| Saint Kitts and Nevis | 1.37 | 1.18–1.67 | 1.46 | 1.17–1.82 | 41.00 | 33.38–52.45 | 41.79 | 30.58–55.40 |
| Saint Lucia | 2.22 | 1.96–2.50 | 2.86 | 2.37–3.46 | 68.19 | 61.48–76.96 | 76.91 | 63.55–93.98 |
| Saint Vincent and the Grenadines | 1.73 | 1.53–1.93 | 1.99 | 1.69–2.31 | 52.86 | 46.01–58.74 | 55.68 | 46.53–65.65 |
| Samoa | 1.64 | 1.05–2.65 | 1.49 | 0.96–2.39 | 62.97 | 39.33–101.80 | 56.25 | 34.40–92.00 |
| San Marino | 3.02 | 2.12–4.09 | 3.27 | 2.22–4.52 | 52.91 | 37.95–71.21 | 55.44 | 37.82–78.76 |
| Sao Tome and Principe | 1.36 | 1.06–1.67 | 1.11 | 0.81–1.46 | 68.64 | 51.59–92.53 | 50.18 | 37.06–68.38 |
| Saudi Arabia | 0.98 | 0.60–1.29 | 0.85 | 0.51–1.25 | 46.42 | 28.97–61.49 | 39.48 | 24.26–57.77 |
| Senegal | 2.07 | 1.48–3.01 | 1.77 | 1.22–2.58 | 109.63 | 78.00–156.30 | 85.03 | 56.69–123.24 |
| Serbia | 0.91 | 0.58–1.08 | 0.86 | 0.53–1.16 | 23.44 | 16.95–27.21 | 21.46 | 14.64–27.79 |
| Seychelles | 0.52 | 0.41–0.59 | 0.54 | 0.40–0.66 | 18.61 | 14.63–21.33 | 18.83 | 14.04–22.84 |
| Sierra Leone | 3.49 | 2.22–5.03 | 2.62 | 1.70–3.77 | 232.79 | 129.42–359.90 | 166.55 | 100.86–252.05 |
| Singapore | 0.27 | 0.21–0.30 | 0.29 | 0.22–0.32 | 8.06 | 6.65–8.86 | 7.61 | 6.26–8.60 |
| Slovakia | 0.68 | 0.44–0.82 | 0.72 | 0.39–1.00 | 19.35 | 13.36–22.79 | 19.89 | 12.30–26.72 |
| Slovenia | 5.18 | 2.30–5.95 | 5.05 | 2.77–6.74 | 111.47 | 59.40–125.79 | 104.59 | 63.56–139.31 |
| Solomon Islands | 3.01 | 2.03–4.64 | 2.85 | 1.96–4.43 | 152.98 | 103.82–236.77 | 141.47 | 95.79–219.52 |
| Somalia | 3.85 | 1.91–8.54 | 3.33 | 1.53–8.65 | 243.63 | 129.77–465.38 | 200.42 | 99.58–459.48 |
| South Africa | 1.42 | 1.20–1.67 | 1.18 | 0.98–1.37 | 60.97 | 51.60–73.06 | 46.99 | 39.13–55.37 |
| South Sudan | 2.15 | 1.42–3.14 | 1.81 | 1.19–2.73 | 145.02 | 90.96–210.25 | 113.16 | 76.26–163.18 |
| Spain | 1.55 | 1.33–1.74 | 1.61 | 1.38–1.95 | 30.76 | 27.60–35.37 | 29.77 | 26.44–35.59 |
| Sri Lanka | 0.96 | 0.68–1.11 | 0.86 | 0.59–1.19 | 31.60 | 22.66–35.91 | 25.98 | 18.20–35.56 |
| Sudan | 1.79 | 0.90–2.86 | 1.29 | 0.66–1.97 | 100.94 | 50.85–155.02 | 67.50 | 35.01–102.17 |
| Suriname | 4.69 | 4.00–5.51 | 5.29 | 4.19–6.54 | 167.83 | 138.57–197.18 | 165.35 | 128.84–207.98 |
| Sweden | 1.96 | 0.97–2.24 | 1.67 | 0.97–1.93 | 35.55 | 25.86–41.34 | 26.14 | 19.45–29.49 |
| Switzerland | 0.86 | 0.59–0.97 | 0.83 | 0.60–0.95 | 17.17 | 13.22–19.39 | 15.90 | 12.38–17.98 |
| Syrian Arab Republic | 0.62 | 0.49–0.75 | 0.88 | 0.59–1.20 | 27.82 | 23.32–34.16 | 31.30 | 23.12–41.37 |
| Taiwan (Province of China) | 0.76 | 0.42–0.85 | 0.97 | 0.51–1.29 | 18.21 | 11.11–20.11 | 21.27 | 12.12–28.21 |
| Tajikistan | 0.39 | 0.28–0.48 | 0.33 | 0.23–0.42 | 24.08 | 16.34–32.04 | 20.32 | 13.97–26.36 |
| Thailand | 0.60 | 0.50–0.78 | 0.61 | 0.44–0.87 | 23.68 | 19.61–28.16 | 20.64 | 15.55–27.53 |
| Timor-Leste | 0.68 | 0.44–1.18 | 0.69 | 0.47–1.17 | 33.19 | 21.40–54.96 | 30.93 | 20.35–49.86 |
| Togo | 1.91 | 1.33–2.51 | 1.56 | 1.11–2.04 | 105.19 | 71.33–145.40 | 76.88 | 53.33–107.64 |
| Tokelau | 1.88 | 1.28–2.91 | 1.47 | 1.01–2.25 | 61.03 | 40.98–96.54 | 49.77 | 32.80–78.46 |
| Tonga | 1.22 | 0.76–2.04 | 1.20 | 0.79–1.95 | 46.05 | 29.04–76.49 | 42.14 | 27.04–67.64 |
| Trinidad and Tobago | 2.71 | 2.20–2.98 | 3.06 | 2.24–4.06 | 79.11 | 64.90–86.29 | 80.61 | 57.93–108.23 |
| Tunisia | 1.11 | 0.62–1.57 | 1.06 | 0.61–1.53 | 43.48 | 24.28–62.47 | 36.80 | 21.33–52.86 |
| Turkey | 0.46 | 0.39–0.58 | 0.47 | 0.37–0.59 | 17.96 | 15.22–21.32 | 16.53 | 13.49–20.13 |
| Turkmenistan | 0.32 | 0.28–0.35 | 0.31 | 0.25–0.38 | 19.98 | 16.24–22.79 | 18.53 | 14.43–22.64 |
| Tuvalu | 2.15 | 1.47–3.25 | 1.96 | 1.36–3.06 | 83.52 | 58.80–126.59 | 73.55 | 51.13–115.29 |
| Uganda | 1.70 | 1.07–2.96 | 1.33 | 0.84–2.35 | 106.89 | 68.81–162.45 | 77.55 | 49.97–120.32 |
| Ukraine | 0.51 | 0.47–0.65 | 0.62 | 0.52–0.76 | 18.25 | 16.75–21.63 | 22.32 | 18.89–26.74 |
| United Arab Emirates | 0.81 | 0.31–1.44 | 0.98 | 0.36–1.85 | 48.81 | 21.94–81.68 | 50.41 | 22.18–90.58 |
| United Kingdom | 1.46 | 1.29–1.65 | 1.88 | 1.45–2.01 | 30.41 | 26.66–32.52 | 38.50 | 28.76–41.12 |
| United Republic of Tanzania | 2.38 | 1.61–3.77 | 1.82 | 1.22–3.08 | 152.11 | 100.49–216.13 | 106.82 | 73.05–159.84 |
| United States of America | 1.37 | 1.25–1.75 | 1.53 | 1.39–1.90 | 51.32 | 43.53–62.56 | 53.83 | 45.34–65.79 |
| United States Virgin Islands | 2.92 | 2.36–3.42 | 3.28 | 2.57–4.03 | 72.83 | 59.55–84.96 | 74.25 | 57.74–92.16 |
| Uruguay | 3.48 | 2.99–3.80 | 3.96 | 3.15–4.43 | 78.26 | 68.96–84.93 | 85.13 | 69.84–94.10 |
| Uzbekistan | 0.29 | 0.26–0.32 | 0.29 | 0.24–0.35 | 15.47 | 13.75–17.59 | 15.48 | 12.82–18.61 |
| Vanuatu | 1.93 | 1.19–3.03 | 1.91 | 1.20–3.01 | 91.91 | 58.27–142.91 | 84.88 | 55.73–134.20 |
| Venezuela (Bolivarian Republic of) | 0.62 | 0.53–1.03 | 0.80 | 0.58–1.32 | 22.77 | 19.97–36.77 | 26.19 | 19.21–41.56 |
| Viet Nam | 0.75 | 0.53–1.22 | 0.73 | 0.46–1.23 | 27.17 | 19.03–43.54 | 23.90 | 15.66–39.95 |
| Yemen | 1.56 | 0.77–2.54 | 1.43 | 0.72–2.33 | 86.84 | 43.31–137.35 | 72.42 | 36.59–117.69 |
| Zambia | 1.95 | 1.46–2.64 | 1.30 | 0.92–1.87 | 121.18 | 85.43–159.79 | 72.73 | 52.35–98.17 |
| Zimbabwe | 1.78 | 1.14–2.31 | 1.62 | 0.98–2.25 | 79.61 | 56.70–102.69 | 73.73 | 48.86–99.78 |

UI, uncertainty interval; DALY, disability adjusted life year
